# Metagenomic analysis of oral microbiota among oral cancer patients and tobacco chewers in Rajasthan, India

**DOI:** 10.6026/97320630018757

**Published:** 2022-09-30

**Authors:** Mukesh Kumar Sharma, Vivek Kumar Srivastav, Chetan Kumar Joshi, Mohan Kumar, K. Manohar Bhat

**Affiliations:** 1 Department of Biotechnology, Maharaj Vinayak Global University, Jaipur Rajasthan, India; 2 Department of Botany, Vishwa Bharti PG College, Sikar, Rajasthan India; 3 Department of Zoology, Government Science College, Sikar, Rajasthan, India; 4 Gyan Joyti College of Pharmacy and Nursing School, Hazaribagh, Jharkhand India; 5 Department of Pedodontics and Preventive Dentistry, Jaipur Dental College, Jaipur, Rajasthan India

**Keywords:** Tobacco chewers, oral cancer, meta-genomics, microorganism, oral

## Abstract

Data on the microbial composition among tobacco chewers and oral cancer patients in Rajasthan, India is of interest. NGS analysis from tobacco chewers and oral cancer comprised the most abundant and core microbial taxa in the oral cavity. It shows that highly pathogenic phylum consisting of 6% Fusobacteria and 9% Firmicutes are observed in oral cancer samples; whereas, 0.6% Treponema, 34% Firmicutes, 0.02% Mollicutes, and 4% Fusobacteria are seen in tobacco chewers. Thus, data shows that the most abundant and core microbial taxa are found in the oral cavity of tobacco chewers and oral cancer patients in Rajasthan, India.

## Background:

Periodontitis and dental caries are the two most prevalent oral diseases and the primary causes of tooth loss in the western world. [1, 2] At present, periodontitis and dental caries are mostly diagnosed at the late stages of the disease, often leading to costly and invasive dental treatment [2]. Therefore, new diagnostic approaches capable of identifying periodontitis and dental caries at preclinical stages, favoring preventive treatment strategies, are urgently needed. The oral cavity harbors a diverse microbiota comprising more than 700 unique bacterial species [3]. The microbiota plays a pivotal role in the maintenance of oral homeostasis, as various oral habitats are colonized by characteristic bacterial community profiles organized in local biofilms [4]. However, ecological changes, for example, increased sugar intake, insufficiently performed oral hygiene or fluctuations in the immune response can induce structural [5,7] and functional alterations [8-10] of local oral biofilms. Such alterations may in turn change the relationship between the host and the resident microbiota from symbiosis to dysbiosis, thereby fueling the initiation and progression of periodontitis and dental caries [7]. Saliva is the biological fluid of the oral cavity which is critical for the maintenance of oral and general health [6]. Therefore, saliva has been intensively investigated for candidate biomarkers associated with oral health and disease [5,8]. Saliva is sterile when entering the oral cavity [9], but when sampled, saliva contains a diverse microbiota [10]. In healthy oral conditions, the composition of the salivary microbiota is different from that of supragingival and subgingival biofilms [8]. On the other hand, the presence of specific bacterial species in saliva such as Porphyromonasgingivalis and Streptococcus mutants has been reported in individuals with periodontitis and dental caries, respectively [9, 10]. Essentially, these findings suggest that bacteria from local periodontitis and caries lesions may be spilledover and dispersed into saliva [10]. However, it remains unclear if dispersed bacteria remain metabolically active as they are translocated from the local ecological niche of the biofilms to saliva, which possesses different ecological properties. So far, only a few studies have reported higher expression of specific bacterial genes associated with dental caries [11,12]. Therefore, it is of interest to document data on the metagenomic analysis of oral microbiota among oral cancer patients and tobacco chewers in Rajasthan, India.

## Methodology:

### Importing the data:

Datasets were imported from different samples (cancer patients and tobacco chewers). The original fastq was converted to fasta. We performed a multi sample analysis with the parent sequence. Optimization of files including the removal of duplicate sequences was completed.

### Quality control:

Dataset was filtered based on length, base quality, and maximum homo-polymer length.

### Sequence alignment:

Aligning sequences to a reference helps improve OTU (Operational Taxonomic Units) assignment [11]. The alignment of sequences to the V4 variable region of the 16S rRNA was completed. This alignment was created as described in [mothur'sMiSeq SOP] from the Silva reference database.

### Extraction of taxonomic information:

We took the sequences and assign them to a taxon. We grouped (or cluster) sequences based on their similarity to defin Operational Taxonomic Units (OTUs): groups of similar sequences that can be treated as a single "genus" or "species" (depending on the clustering threshold). The first step is to further de-noise our sequences from potential sequencing errors, by pre-clustering the sequences and classifying the sequences using a training set, which is again provided on [mothur'sMiSeq SOP]. The next step is to use this information to determine the abundances of the different taxa. This consists of three steps: (i) first, all individual sequences are classified and assigned a confidence score (0-100%); (ii) Next, sequences are grouped at a 97% identity threshold (not using taxonomy data); (iii) finally, for each cluster, a consensus classification is determined based on the classification of the individual sequences taking their confidence scores into account.

### Visualization:

We visualized results is an HTML file with an interactive visualization tool.

## Results and Discussion:

### Sequence data statistics

Post sequencing trimming and quality control of raw sequences from sequencing of samples - control, tobacco chewers, and oral cancer resulted from reads are mentioned in Table 1(see PDF). Data from Sequence Read Archive (SRA) database under Bio project Accession number PRJNA751046 is used. 

Reads from Tobacco chewer's samples combined to contig resulted in 117721 sequence reads at an average sampling of approximately 39240 reads optimized to remove duplicate sequences to 63191. Moreover, oral cancer samples resulted in 243766 sequence reads with an average sampling of approximately 81255 reads, optimized to 103014 sequence reads. A total of 119584 sequence reads were observed with an average sampling of 39861 optimized to 62057 sequence reads in control samples. Negative controls generated minimal sequence data that were not included in the analysis. After alignment by using the Mothur package in Galaxy software, TC unique representative sequences were classified where 32214 into 5899 operational taxonomic units (OTUs) with 60% confidence percentage cutoff at a 97% similarity level using average neighbor clustering method and the distance threshold is 0.15. Moreover, OC samples classified 50860 sequences into 9788 OTUs with a 96.2% similarity level. However, 30565 control sample sequences were observed with 5895 OTUs.

### Oral microbiome composition of patients with oral cancer and tobacco chewers:

The bacterial communities in the cancer samples and the matched controls clustered separately, suggesting the overall structures of the bacterial communities in the groups were significantly different. Metagenomic data revealed a relative abundance of microbial communities in all three sample types that illustrate a higher abundance of known oral pathogens. TC microbial composition was higher in known and opportunistic oral pathogens having a decreased amount of known oral commensal bacteria when compared with OC. Taxonomic analysis revealed in OC and TC samples (Figure 2, Figure 3 - see PDF) that a substantial percentage of sequence data belonging to genera is known to contain pathogens or opportunistic oral pathogens. The sequence data showed that the microbial composition varied between TC and OC (Figure 4 and Figure 5 - see PDF). We determined that the microbial composition associated with tobacco chewers and oral cancer was unusual. Sequences from TC and OC comprised the most abundant and core microbial taxa among the three sample types revealing three discernable communities in the oral cavity. From classification, we observed the abundance of phylum with 34% Bacteroidetes, 34% Firmicutes, 21% Proteobacteria, and 4% Actinobacteria in the TC oral cavity in contrast 34% Bacteroidetes, 41% Proteobacteria, and 4% Actinobacteria to OC. Highly pathogenic phylum i.e. 6% Fusobacteria, 9% Firmicutes observed in oral cancer samples whereas in tobacco chewers samples comprised 0.6% Treponema, 34% Firmicutes, 0.02% Mollicutes and 4% Fusobacteria. Several sequences are unclassified under OTUs. The pie chart analysis shows that the most abundant and core microbial taxa between the three sample type's revealed three discernable communities in the oral cavity.

TC microbial composition was higher in abundance of known and opportunistic oral pathogens while having a decreased amount of known oral commensal bacteria when compared with OC. Taxonomic analysis revealed that a substantial percentage of sequence data belonging to genera known to contain oral pathogens or opportunistic oral pathogens such as Gemella Species 2% (gram-positive bacteria), Treponema 0.6% (spirochaete bacterium), Erysipelotrichaceae 0.4% (Firmicutes), Gamma proteo-bacteria 12%, Betaproteobacteria 7%, Campylobacteria 0.5%, Coriobacteriaceae 0.3% (Actinobacteria), Fusobacteria 4% was present in TC. Species pathogenic in nature or opportunistic pathogen with Operational Taxonomy Unit are listed in Table 2(see PDF). Moreover, Taxonomic analysis unfolds the sequences classified to contain pathogens with OTU quantity such as Erysipelotrichaceae 0.2% (Firmicutes), Leptotrichia 2% (Fusobacteria),Betaproteobacteria 34%, Campylobacteraceae 0.2% in oral cancer listed in Table 3(see PDF).

Reads from tobacco chewers samples combined to contig resulted in 117721 sequence reads and an average sampling of approximately 39240 reads and optimized to remove duplicate sequences to 63191, moreover, oral cancer samples resulted in 243766 sequence reads with average sampling of approximately 81255 reads, optimized to 103014 sequence reads. A total of 119584 sequence reads were observed with average sampling of 39861 and optimized to 62057 sequence reads in Control samples. Negative controls generated minimal sequence data and were not included in our analysis.

After alignment using Mothur package in Galaxy software, TC unique representative 32214 sequences were classified into 5899 operational taxonomic units (OTUs) with 60% confidence percentage cutoff at 97% similarity level using average neighbor clustering method and the distance threshold is 0.15. Moreover, OC samples classified 50860 sequences into 9788 OTUs with a 96.2% similarity level. However, 30565 control samples sequences were observed with 5895 OTUs. Compared with other studies, our study identified larger number of distinguishing taxa at each level using the LEfSe method. At the phylum level, Firmicutes and Actinobacteria presented with the same patterns reported by Schmidt [13] and were remarkably decreased in cancer lesions, while significant increases in Fusobacteria was also observed, consistently [14].

Genera Streptococcus and Rothia were significantly decreased in cancer lesions as reported elsewhere [13-14]. Majority of these significantly enriched genera in lesions are involved in periodontal disease, including Fusobacterium, Dialister, Peptostreptococcus, Filifactor, Peptococcus, Catonella, and Parvimonas [15]. Consistent with previous findings, remarkable enrichment of Peptostreptococcus and Parvimonas was observed in cancer samples [16, 17]. Additionally, Veillonella was significantly decreased in cancer lesions, a finding that was previously reported in 73% of oral cancer patients after treatment [16], indicating Veillonella correlates with a healthy status. Of the distinguishing species identified across the groups, forty species were highly abundant in cancer lesions, including Porphyromonasen dodontalis, Filifa toralocis and Dialister pneumosintes, which are newly, recognized periodontal pathogens [17]. Of all oral bacteria, Porphyromonas gingivalis and Fusobacterium nucleatum possess the greatest potential to be correlated with oral cancer, as both have been implicated in pancreatic and colorectal cancers. Recently, a report by Gallimidi showed P. gingivalis and F. nucleatum promote oral cancer progression via direct interactions with oral epithelial cells through Toll-like receptors [18]. However, P. gingivalis did not differ in abundance between groups. Fusobacterium, comprising the species periodonticum, naviforme, and nucleatum_subsp, was significantly enriched in lesions, accounting for 8.33%, 0.103%, and 0.297% of sequences in the cancer group, respectively. F. periodonticum, F. naviforme, and F. nucleatum_subsp were reported to account for 4.08%, 0.01% and 11.67% of sequences in cancer samples, respectively [14]. Thus, the different prevalence of Fusobacterium species detected in OSCC samples between studies may largely be due to differences in sample types, races and geographic regions of the subjects recruited. Further evidence is needed to verify these findings. A higher abundance of several Treponema species was observed in cancer lesions. T. denticola, a member of the periodontal “red complex” involved in pancreatic cancer [19], was not included. In the literature, Bacteroides fragilis has been linked to colon cancer [20], but it was not observed in our study, although it was detected in OSCC tissues in another report [21]. Capnocytophaga levels were significantly higher in the saliva of lung cancer patients [22] than in healthy controls, and Capnocytophaga gingivalis was previously suggested to be a potential salivary biomarker of oral cancer [21]. In this study, C. gingivalis was detected at higher levels in control samples without any significance, while C. leadbetteri and C. sp_oral_taxon_902 were remarkably overabundant in lesions. Members of the genus Selenomonas have been repeatedly associated with periodontal disease, although the Selenomonas species detected in this study did not correlate with known diseases [19]. Several species of Peptostreptococcus and Parvimonas were extensively enriched in cancer samples, including Peptostreptococcus stomatis and Parvimonasmicra, both of which are reportedly related to colorectal cancer [23]. Eikenellacor rodens, a fastidious gram-negative facultative anaerobic bacillus, was also detected in another study [14]. The genus Eikenella is significantly overrepresented in colorectal cancer [4] and is associated with HPV-negative head and neck squamous cell carcinoma samples [17]. Given its documented history of pathogenicity, further investigation of the potential role of E. corrodens in the etiology of OSCC is warranted. In our design, paired lesion and control samples were procured from one individual, eliminating inter-individual variation. Therefore, even slight differences in the bacterial profiles between groups may be closely correlated with OSCC. Although several of the distinguishing taxa were present in relatively tiny proportions, their role in the development of OSCC should not be ignored. Bacteria coexist in complex interaction webs, and interactions within these webs affect the species involved, while perturbations may contribute to disease. As shown in network analysis, bacterial communities in OSCC samples presented with more complex webs depicting ecological relationships, consistent with the extensive bacterial diversity detected in the samples. The genera Prevotella and Neisseria clustered, forming two of the densest interaction webs in both groups. Prevotella and Neisseria play key roles in maintaining the stability of the oral bacterial community across samples. Conversely, an association networkk centered around Fusobacterium arose in the cancer group, indicating that the genus Fusobacterium was implicated in the development of OSCC following its significant increase in the cancer group. Fusobacterium tends to co-adhere with other species in oral biofilms by forming bridges between early and late colonizers. Thus, it was reasonable to infer a critical role for Fusobacterium in increasing OSCC bacterial diversity. Further evaluation of the role of Fusobacterium in OSCC is required. It was observed that the same paired taxa showed absolute opponent relationships within the groups, implicating that some drastic changes in the bacterial symbiotic relationships occurred during the oral carcinogenesis.

Smokers had significant increase in Prevotella and Capnocytophaga and decreased Granulicatella, Staphylococcus, Peptostreptococcus, and Gemella when compared to the other two groups. A significant decrease in the abundance of Peptostreptococcus in smokers has been evidenced before [24], suggesting the susceptibility of this genus to smoke exposure. It is of interest to note that this particular reduction may be significant as several species belonging to this genus have shown to interfere in the growth of pathogenic bacteria in the upper respiratory tract [25]. Another genus that also seems to be modulated by smoking is Gemella, with a previous study also finding a decrease in the abundance of this genus [26]. In our analysis, the genus Porphyromonas, which is increased in smokers [26, 27] and has a role in periodontitis [25,27], was also found to have higher abundances in only smokers. Subsequent reports using 16S rRNA sequence profiling of subgingival plaque identified an increase in several disease-associated organisms in smokers, including Parvimonas, Fusobacterium, Campylobacter, Bacteroides, Dialister, and Treponema spp. and a decrease in potential health-promoting taxa from the Veillonella, Neisseria, Streptococcus, and Capnocytophaga genera [28]. Capnocytophaga, Fusobacterium, and Neisseria in the oropharynx of smokers [27] and alterations in 172 subgingival plaque OTUs in smokers is known [29]. Because of the various sample types used to study the oral microbiome, and the known variation in microbial communities in different parts of the oral cavity [30], comparison across studies is difficult. Several microbes have been reported as direct or indirect triggers for CD progression.

## Conclusions:

We document preliminary information from a meta-genomics using next-generation sequencing technologies that has produced bacterial profiles and genomic profiles to show the relationships between microbial diversity, genetic variation, and oral diseases. An abundance of specific oral bacterial species in the oral microbiome of patients with oral cancer and tobacco chewers is observed.
